# Brain and Thought

**Published:** 1854-12

**Authors:** 


### BRAIN AND THOUGHT.

Richmond mentions the case of a woman whose brain was exposed in consequence of the removal of a considerable portion of its bony covering by disease. He says he repeatedly made pressure on the brain, and each time suspended all feelings and all intellect, which were instantly restored when the pressure was withdrawn. The same writer also relates another case, that of a man who had been trepanned, and who perceived his intellectual faculties failing, and his existence drawing to a close, every time the effused blood collected upon the brain so as to produce pressure.

Professor Chapman, of Philadelphia, mentions, in his lectures, that he saw an individual with his skull perforated, and the brain exposed, who was accustomed to submit himself to the same experiment of pressure as the above, and who was exhibited by the late Professor Westar to his class. His intellectual and moral faculties disappeared on the application of pressure to the brain; they were held under the thumb, as it were, and restored at pleasure to their full activity by discontinuing the pressure. But the most extraordinary case of this kind within my knowledge, and one peculiarly interesting to the physiologist and metaphysician, is related by Sir Astley Cooper in his surgical lectures.

A man by the name of Jones received an injury on his head, while on board a vessel in the Mediterranean, which rendered him insensible. The vessel, soon after this, made Gibraltar, where Jones was placed in the hospital, and remained several months in the same insensible state. He was then carried on board the Dolphin frigate to Deptford, and from thence was sent to St. Thomas's Hospital, London. He lay constantly upon his back, and breathed with difficulty. His pulse was regular, and each time it beat he moved his fingers. When hungry or thirsty, he moved his lips and tongue. Mr. Clyne, the surgeon, found a portion of the skull depressed, trepanned him, and removed the depressed portion. Immediately after this operation the motion of his fingers ceased, and at four o'clock in the afternoon (the operation having been performed at one) he sat up in bed; sensation and volition returned, and in four days he got out of bed and conversed. The last thing he remembered was the circumstance of taking a prize in the Mediterranean. From the moment of the accident, thirteen months and a few days, oblivion had come over him, and all recollection ceased. He had for more than one year drank of the cup of Lethe, and lived wholly unconscious of existence; yet, on removing a small portion of bone which pressed upon the brain, he was restored to the full possession of the powers of his mind and body.

